# Idiopathic Hypersomnia—A Dynamic Simulation Model

**DOI:** 10.3389/fneur.2022.902637

**Published:** 2022-06-10

**Authors:** Marek Susta, Karel Šonka, Gustav Bizik, Svojmil Petranek, Sona Nevsimalova

**Affiliations:** ^1^Department of Public Health, St. Elisabeth University, Bratislava, Slovakia; ^2^Department of Neurology, First Faculty of Medicine, Charles University and General University Hospital, Prague, Czechia; ^3^Department of Psychiatry, Aalborg University, Aalborg, Denmark; ^4^Health Care Facility, Department of the Interior, Prague, Czechia

**Keywords:** idiopathic hypersomnia, dynamic modeling, treatment strategy, sleep disorders, work impairment

## Abstract

**Aims of the study:**

Commonly used approach to illness assessment focuses on the patient's actual state supplemented by binary records of past events and conditions. This research project was designed to explain subjective experience in idiopathic hypersomnia (IH) patients influenced by their clinical symptoms and comorbidities.

**Material and Methods:**

Forty-three IH patients of both sexes (female 60.5%, male 39.5%) were assessed using a detailed structured examination. The interview covered neurologic, psychiatric, and internal medicine anamnesis, medication past and current, substance abuse, work impairment, detailed sleep-related data, specific sleep medication, and a full-length set of questionnaires including depression, quality of life, sleepiness, anxiety, fatigue, insomnia, and sleep inertia. The data were digitized and imported into statistical software (SPSS by IBM), and dynamic simulation software (Vensim by Ventana Systems Inc.) was used to build a causal loop diagram and stocks and flows diagram as a simulation structure.

**Results:**

The overall raw data and simulation-based patterns fit at 76.1%. The simulation results also identified the parameters that contribute the most to patients' subjective experience. These included sleep inertia, the refreshing potential of naps, the quality of nocturnal sleep, and the social aspects of the patient's life. Psychiatric disorders influence the overall pattern at a surprisingly low level. The influence of medication has been studied in detail. Although its contribution to the dynamics looks marginal at first sight, it significantly influences the contribution of other variables to the overall patient experience of the disease.

**Conclusion:**

Even the simplified dynamic structure designed by the research team reflects the real-life events in patients with IH at the acceptable level of 76.1% and suggests that a similar structure plays an important role in the course of the disease. Therapeutic focus on the parameters identified by the model should enhance the patients' subjective experience throughout illness duration and might even turn the progress from negative into positive. Further research is needed to understand the dynamics of idiopathic hypersomnia in greater detail to better understand the causes and design therapeutic approaches to improve patients' quality of life.

## Introduction

Hypersomnias of central origin are of interest to clinicians and research groups for many reasons. The as-yet unresolved etiology and frequent resistance to therapeutic interventions pose a challenge for both groups (1). From the patient's point of view, it is a disorder with a significant impact on quality of life, and from society's point of view, sleep-wake disorders are a significant and, according to current research, an ever-increasing source of costs (2). Therefore, advances in understanding the disease's pathophysiology and therapeutic approaches are eagerly awaited. Most of the research in the field of hypersomnias is devoted to narcolepsy type 1 (NT1); this project focused on idiopathic hypersomnia (IH) (3). This disorder receives a great deal of attention in the neurological department of the First Faculty of Medicine and General University Hospital because it was there when Bedrich Roth first described the concept of “sleep drunkenness” in the 1950s (4). Unfortunately, reliable data on the incidence and prevalence of this disease remains unknown; some sources report that the number of patients is about one-third that of NT1 (5). Diagnosis of the disease is quite complex, and the technical and logistical requirements include repeated testing with polysomnography and the Multiple Sleep Latency Test (MSLT) (6). The current third version of the International Classification of Sleep Disorders requires that other possible causes of sleep disturbance, including psychiatric, drug-induced, or drug abuse, be excluded for diagnosis. The search for a marker typical of IH has not yet been successful, and sleep drunkenness, reported in many papers as a prominent disease feature in the latest version of the classification, is not even listed among the criteria (7).

Parameters of excessive sleepiness affecting the experience of illness have been studied in detail in various contexts and can be found in the relevant literature. The phenomena that positively and negatively affect the experience are also the subject of therapeutic approaches (8). For IH patients, these include parameters related to fatigue and sleepiness, sleep quality and duration, post-awakening state, and associated social, occupational, and personal difficulties. In general, the experience is also influenced by other diseases, both psychiatric and somatic. The issue of the validity of patient testimony has been addressed in many papers (9, 10), and in general, tools based on the personal testimony of patients are considered reliable. In addition, our project was, without exception, a re-evaluation of the diagnosis in patients whose clinical status was known throughout the time since diagnosis.

Although IH is commonly described as a chronic disease, the diagnostic criteria are based on assessing a single time point and include the time criterion only indirectly (as is the case, for example, with psychiatric conditions in the exclusion criteria). However, in terms of the impact of IH on patients' quality of life and overall function, a temporal characteristic, including the duration of the illness and the dynamics of the illness in interaction with clinical and non-clinical factors maintaining and preventing the illness is essential. To the best of our knowledge, this central aspect of IH has not yet been systematically studied, but modeling and simulation tools have proven helpful in describing the dynamics of specific clinical features of sleep disorders. Other research teams have constructed models to explain certain aspects of hypersomnia. In particular, a model of fatigue after traumatic brain injury (11), modeling the dimensions of Excessive Daytime Sleepiness using linear methods (12), or simulating neuronal ensembles in the search for causes of sleepiness (13). Drawing on clinical medical research experience and expertise in systems science and computational modeling, our team designed this project to overcome methodological challenges that require an interdisciplinary approach. The project combines a large database of patients with IH with a large set of relevant clinical and non-clinical data with their time course and, based on these inputs, develops a dynamic model that proposes an underlying dynamic structure suitable for rigorous testing, determines the relative contribution of each parameter to patient-reported disease dynamics, and suggests targets for rational therapeutic interventions and future research.

## Participants and Methods

### Study Participants

All study participants (17 males and 26 females) were diagnosed with idiopathic hypersomnia according to the International Classification of Sleep Disorders version 2 or 3 criteria (7, 14). As a result, the initial number of patients dropped from 46 to 43. For the three excluded patients, data analysis raised doubts about whether the diagnostic work-up was completely accomplished. The basic sleep statistics of the cohort are shown in Table 1. Proportions of patients with ESS >10 at the time of diagnosis 85%, total sleep >660 min/24 h 78.6%, and MSLT <8 min 73.8%.

**Table 1:** Basic age and sleep characteristics of the participants.

	**Min-max**	**Median**	**Inter-quartile range**	**95% confidence intervals**	
				**Lower bound**	**Upper bound**
Age in interview (years)	20–67	43	19.5	38.6	46.1
Age of illness onset (years)	6–52	18	20.5	20.8	28.9
ESS in onset	6–23	15	6	13.1	16.1
MSLT latency (min.)	1–15.9	5.2	4.3	5.1	6.9
MSLT SOREM	0–1	0	0	-	-
24 h sleep length (min.)	603–1,100	690.5	99.7	674.9	754

### Specialized Structured IH Interview and Data Gathering

A specialized structured interview with IH patients was developed for the project, which included complete anamnestic data, work and education history, experience, family history of sleep disorders, neuropsychiatric history, somatic illnesses, past, and current medication, substance abuse, and complete sleep features. In addition, anamnestic data were enriched by questionnaires covering psychiatric (Beck Depression Inventory-II, State Trait Anxiety Inventory), social (Quality of Life), neurological (Pain), and sleep (Circadian preference, Epworth Sleepiness Scale, Fatigue Severity Scale, Insomnia Severity Scale, Sleep Inertia) domains. The evaluation included differential diagnosis to exclude DSPS or other possible hypersomnias. Patients were examined by PSG, MSLT, and submitted sleep diary and actigraphy records. Of the 43 patients, 39 (90%) accepted to undergo HLA-DQB1^*^06:02 phenotyping, 9 (20%) with positive results. DSPS was excluded by repeated careful history taking and by actigraphy and sleep diary performed before PSG and by PSG itself. Head and cervical spine MRI was evaluated to exclude structural lesions. Of all the structured interview items, 71 were formulated as model input parameters, and so great attention was paid to their accuracy and completeness (15).

Sleep parameters served in extenso as input parameters for the model, and data on other neurological and somatic diseases were aggregated. As the department is part of a university hospital that covers all medical specialties, the information system allows the diagnostic findings of other departments to be displayed. Non-neurological comorbidities were entered into the questionnaire based on medical reports from the patient's documentation. The comorbidities were carefully checked and, if necessary, documented by reports by other physicians. The comorbidities were divided into sleep, psychiatric and other comorbidities. Sleep comorbidities were included individually in the model, and non-sleep comorbidities were collected into system groups. The prevalence of some of the diseases studied was not high in the cohort; endocrinological diseases occurred in four patients, epilepsy in three, neuroimmunological diseases in three, malignant tumors in three, a cognitive deficit in one, diabetes in one, and rheumatoid arthritis in one. Ischemic or hemorrhagic stroke, multiple sclerosis, synucleinopathy, and tauopathy did not occur at all. The frequencies of more common disorders and diseases potentially related to hypersomnias are shown in Table 2.

**Table 2:** Frequency of more common disorders and diseases potentially related to hypersomnias within the cohort.

	**Frequency**	**%**
Headaches	21	52.3
Vertebrogenous	21	47.7
Gastroininestinal	16	36.4
Depression	12	27.3
Serious infections	12	27.3
Obesity	10	22.7
Hypertension	9	20.5
Cardiovascular	9	20.5
Autoimmune except CNS	9	20.5
Other psychiatric disorders	8	18.2
Thyreopathy	8	18.2
Respiratory	8	18.2
Urologic	8	18.2
Vegetative	7	15.9
Anxiety	6	13.6
Inflamatory CNS	5	11.4

A common part of the clinical examination, and thus also of our dataset, is the question about the present status. Tools have been developed to investigate the disease not only from a strictly medical perspective but also from its occupational, social, and personal aspects (16, 17). However, our structured interview was designed to obtain data for re-evaluation of the diagnosis and capture the dynamics of the evolution of the studied main clinical parameters. To mathematize the process of parameter evolution, it was necessary to obtain data on the beginning of the occurrence and the eventual end of the event of the phenomenon, the extreme values, and the data pattern changes. These were obtained for all parameters listed in output Table 4, which were treated as dynamic. On the other hand, some model input parameters were treated as static, and it was not necessary to obtain data expressing dynamics. Examples include the now obsolete binary parameter IH with a normal length of night sleep/IH with long night sleep or year of disease onset, year of diagnosis, etc.

An unconventional item was a question on the personal experience of the disease evolution and its consequences, in which a verbal response from a predefined set of possible answers (e.g., it is getting worse, it is getting better, the condition is slightly oscillating, the condition is significantly oscillating, etc.,) was requested about halfway through the interview. At the end of the interview, the patient was asked to select the one that best matched their own experience of the disease from a set of nine predefined time-based plotted patterns. A blank graph was also provided to plot the experience that did not match any prearranged patterns. However, the patient's response to the disease experience should be considered retrospective. Authors are aware of a possible bias based on the general human tendency to judge the past by the present, so we asked the question on experience twice. Given the already significant burden on the patient from a long interview, it seems that asking the same question in two different ways and expecting a similar answer was one of the few ways to minimize bias. As these were, without exception, long-term patients, it was possible to derive the response from the documentation, but the aim of the project was to model the personal experience from the personal account, and so the above study design was ultimately chosen.

### Model Construction and Simulation

Since the construction of dynamic models may not be a well-known method, in Table 3, we present the procedure for their construction using a system-dynamic approach.

**Table 3:** Procedure of dynamic model creation and simulation.

**Step**	**Comment**
Identification of variables	Expert consensus based on a set of prominent routinely assessed clinical variables
	The variable can be scalar (single value) or vector (multiple values) type. Example of scalar is patient's age, example of vector is “Other somatic pathologies”
Construction of causal-loop diagram (CLD)	Based on the assumed existence of a relationship between variables that is either scientifically validated and documented (e.g., depression and quality of nighttime sleep) or self-evident (expected specific effect of stimulants on sleepiness), or based on systems analysis and expert consensus.
	“Causal” in models of complex non-linear systems does not refer to causality in a common meaning of the word.
Construction of stock and flow diagram	Step-by-step process based on the system-dynamic methodology, mathematization of variables, relations and their notation in the form of a system of differential equations.
Setting of delays	The impact of changes is delayed in dynamic systems, the length and type of delay is set according to the input data received.
Normalization of input data	Input data were acquired in various units, to be comparable they must be converted to a common scale.
Setting of initial values	The normalized data are inserted into a system of differential equations and the model becomes simulation-ready.
Simulation	The output of the simulation is obtained by solving a system of differential equations in a given sequence of steps representing the time from the onset of the disease to the time of the interview.

The data obtained in the interview were used to construct a dynamic simulation model to answer the question of whether the patient-reported course of the disease can be explained by the dynamics of the other parameters obtained from the structured interview. Our research project used the system dynamics methodology, which uses a system of differential equations to capture the dynamics of the phenomena under study (18, 19). System dynamics have been used repeatedly to describe complex systems and model sleep. Unlike commonly used methods, it allows the inclusion of feedback, nonlinear relationships, time-varying delays, and soft variables (20, 21).

Obtaining the time dimension of the investigated parameters allows for a retrospective reconstruction of their dynamics. If the beginning and the end of the occurrence of a phenomenon and its maximum value is known, its time course can be expressed as the difference of factors increasing and decreasing the value in a given time period. The value of the parameter is then given by a definite integral:


$$PV=\int_{T_{0}}^{T_{f}}\left(IF-DF\right)dt+IV_{T_{0}}\;$$

where *PV* is the parameter's value, *IF* is the sum of the influences increasing the parameter's value, *DF* are the factors decreasing the value, and *IV* is the initial value at the beginning of the period under study.

The data obtained were used to construct a dynamic simulation model to answer the question of whether the patient-reported course of the disease can be explained by the dynamics of the other parameters obtained from the structured interview. Since the patient answered the question of experience with the disease twice, we first compared the consistency of the two answers. Comparison of statements with records differed in only two cases. The model assumes that the resulting experience is determined by the summation of factors with presumed increasing and decreasing impact. Thus, by simulating all parameters and summing them over time, we obtain the overall experience, which can be compared with the patient's testimony. If both patterns match, it can then be argued that the interaction of the given parameters determines the evolution of the patient experience.

All queries for non-descriptive (dynamic) parameters had a uniform structure consisting of time of first occurrence, maximum and minimum values, time of the last occurrence (if any), and parameters affecting state change, i.e., date and rate of change. The negative feedback target-seeking loop principle is described in Section Statistical Analysis of Outcome, and the above parameters constituted the so-called target value to which the level of the parameter gradually approached.

### Statistical/Mathematical Method

General systems theory implies that systems exhibit a finite number of behavioral patterns. Their value may increase, decrease, remain constant or oscillate with constant or variable frequency or amplitude. In addition, complex systems exhibit a phenomenon of dominance shift, after which the original behavior changes from one pattern to another (22, 23). The initially increasing parameter reaches an equilibrium state or starts to increase or decrease. The system's behavior is determined by its structure, expressed by a set of differential equations, and the system's initial state is determined by the values of the exogenous variables (24). The data obtained in the structured interview was used to create the structure of the dynamic simulation model and set its initial values. For example, the evolution of sleepiness can be described by a negative feedback loop whose dynamics is determined by the temporal change in the target value from the disease onset to the state at the time of the structured interview. All parameters of the modeled system can be captured similarly. Some of the parameters affect the experience positively, others negatively. We hypothesize that the sum of all captured parameters should correspond to the patient's reported overall disease experience. However, the captured data cannot simply be summed because they do not have common units. Therefore, the parameters used must be first normalized and converted to a common scale. All continuous, ordinal, and nominal parameters were converted to values in the interval <0;100>. The normalized input data, represented by 71 parameters, enter the model *in extenso*, but the resulting state variables are their logical aggregation. This means that, for example, other pathologies are modeled to the extent described in the structured interview, but the simulation results in an overall pattern called “other pathologies.” Each of the output parameters listed in Table 3 is expressed in the model as a state variable described by an integral whose value at each time step of the simulation is affected by the rate of change represented by the following equation:


$$\delta IF_{t_{n}}=MIN\left(\frac{g_{t_{n}}-PV_{t_{n}}}{d_{t_{n}}},g_{t_{n}}\right)$$

where δ*IF*_*tn*_ is the value of the state variable input, *MIN* is a function that selects the smaller value from the arguments, *g*_*tn*_ is the target value, *PV*_*tn*_ is the value of the state variable, and *d*_*tn*_ is the value of the delay at a given time step. The target can generally take values from < −100;100>. For some variables, it is exclusively in the negative range (e.g., depression), while for others, it can take on both positive and negative values depending on the input data. For example, naps may have a positive value if the patient perceived them as refreshing at the time, or they may be negative if they do not bring refreshment. The model also captures situations where, for example, naps are refreshing but possibly followed by sleep inertia. The delay can also be called the adjustment time; it varies between parameters and determines the rate of change of the value of the state variable. The target changes for each parameter, and its value is the result of the input data dynamization that may change over time, for example, the target effect of a medication is modeled as depending on the effect at the time the medication is administered, and the effect persists only a short while after discontinuation. Modeling the value of the outcome state variable, i.e., the patient's experience of the disease, was the target of a negative feedback loop whose value was given by the sum of the values of all state variables at a given time step. The resulting time course was then compared with the patient's report of disease experience. The output of the dynamic simulation model is temporal data whose frequency is determined by the size of the simulation step. In the presented model, the simulation step is one year and the simulated period is the time from disease onset to the year in which the re-evaluation of the IH diagnosis took place.

**Figure 1:**
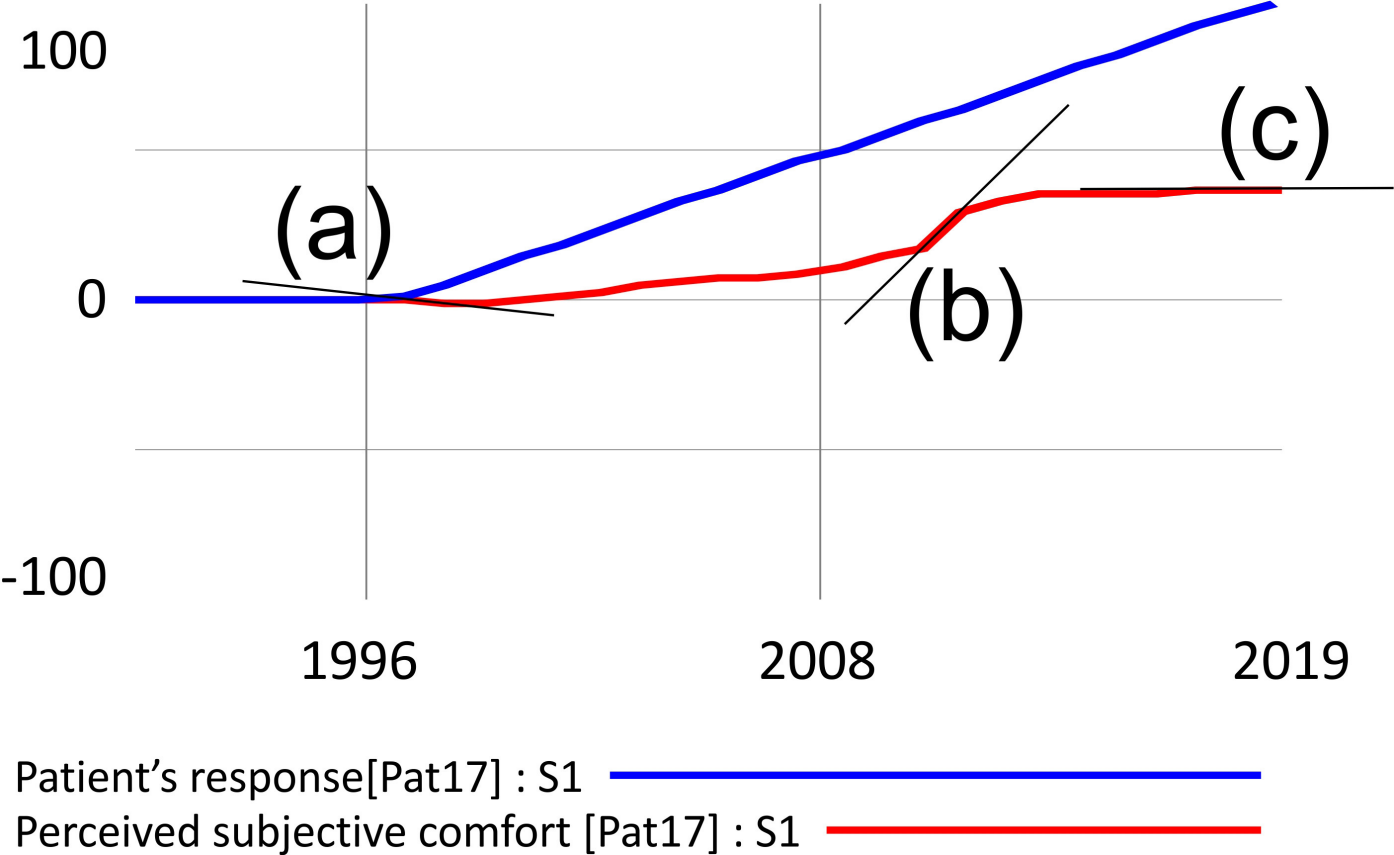
Example of simulation output processing with three possible derivation values. The decreasing function is denoted by **(a)** increasing by **(b)** and constant by **(c)**.

There would be a risk of over fitting if the role of the model was to search for dynamics that best match the predicted behavior (in this case, the reported patient experience). However, this is not the case in the presented model; the structures that model the resulting behavior are entirely independent of the structure that models the patient's response. This is expressed in Figure 1, where the blue and red waveforms arise independently of each other, and the blue waveform is merely a reconstruction of the patient's graphical response. Similarly, for the statistical treatment of the overall results, the design does not risk a type 1 error because the model simulates all cases simultaneously, but each in its own independent domain.

### Statistical Analysis of Outcome

Analysis of the waveforms was performed by comparing the derivation of the two waveforms at all points in the simulated period.

Segments in Figure 1 where the derivation is negative indicate a decrease in the value of the function, denoted as (a). A zero derivation expresses a constant waveform (b), and a positive derivation value shows an increasing function (b). Visual inspection of the waveform can be misleading in some cases. For example, the penultimate simulated segment gives the impression of a constant waveform, but the data analysis showed a positive derivation and, therefore, growth. For instance, if the patient reported that his condition has improved steadily since the onset of the disease, the simulation matches his response in segments where the function expressing the subjective experience is increasing. In the case shown in Figure 1, the match of the simulated course with the patient's response is 89.3%.

The individual simulation results of all study participants were summarized to determine the contribution of each parameter to the overall dynamics. Although 71 parameters entered the model, the output consists of the values of the aggregate levels described in the previous paragraph and shown in Table 3. For example, the values of the output parameter “Somatic pathologies” are given by the dynamics influenced by 24 input parameters, “Naps” by six, etc. Three statistical analyses were performed to assess the contribution of each parameter to the overall patient experience of the disease. The columns show the individual scenarios. Baseline means the entire disease duration for all patients. The “Last 5 years” covers only the output dynamics of the last 5 years of disease for all patients, and “Medicated only” shows the results of patients who were put on medication during the previous 5 years regardless of the clinical outcome. Statistical evaluation of the results was performed using the non-parametric Mann–Whitney test at the 95% confidence level.

## Results

Comparing the simulation output values of patient experience and patient-reported course, there was a fit in 76.1% of patterns. However, this figure does not indicate the number of patients whose simulated and reported patterns did not differ.

Three summary scenarios are presented in Table 4. The first one, presented in the left column, labeled “Baseline,” is the sum of all patient runs over the entire disease duration in the form of the value of the indices (Simulation output SUM) and the absolute value of the contribution of each output item to the overall dynamics (Absolute contribution %). In the second scenario, only the last 5 years of disease duration were considered, and in the last one, shown in the right column, the simulation results display only patients in whom stimulants (methylphenidate or modafinil) were deployed, regardless of the treatment effect. Finally, the last column (*p*) shows the result of the Mann–Whitney test at the 95% significance level, comparing the “Last 5 years” and “Medicated only” scenarios.

**Table 4:** Simulation results, table values description in the results paragraph.

**Model parameter**	**Baseline (*****N =*** **43)**		**Last 5 years (*****N =*** **40)**		**Medicated only (*****N =*** **33)**		***p***
	**Simulation**	**Absolute**	**Simulation**	**Absolute**	**Simulation**	**Absolute**	
	**output SUM**	**contribution %**	**output SUM**	**contribution %**	**output SUM**	**contribution %**	
	**(dimensionless)**		**(dimensionless)**		**(dimensionless)**		
Anxiety	−5,264, 94	4, 6%	−7,695, 29	5, 1%	−2,127, 76	6, 3%	0.111
Depression	−1,468, 65	1, 3%	−1,936, 36	1, 3%	−519, 98	1, 5%	0.345
Sleep inertia	−23,785, 71	20, 7%	−31,467, 42	20, 8%	−7,561, 71	22, 3%	0.48
Work and social	−19,854, 40	17, 3%	−22,906, 08	15, 2%	−2,865, 86	8, 5%	0.004
impairment
Sleepiness	−12,983, 30	11, 3%	−17,127, 30	11, 3%	−3,760, 46	11, 1%	0.028
Fatigue	−5,885, 17	5, 1%	−9,196, 22	6, 1%	−3,168, 03	9, 3%	0.345
Naps	19,689, 76	17, 2%	26,442, 19	17, 5%	5,941, 81	17, 5%	0.028
Nocturnal sleep	17,099, 39	14, 9%	22,418, 76	14, 8%	4,535, 73	13, 4%	0.008
Other psychiatric	−1,502, 53	1, 3%	−1,954, 41	1, 3%	−550, 40	1, 6%	0.345
disorders
Somatic pathologies	−4,773, 05	4, 2%	−5,924, 73	3, 9%	−1,077, 70	3, 2%	0.004
Methylphenidate	1,109, 83	1, 0%	1,768, 80	1, 2%	782, 93	2, 3%	0.421
effect
Modafinil effect	1,254, 81	1, 1%	2,244, 32	1, 5%	1,014, 49	3, 0%	0.5
Sum %		100%		100, 0%		100, 0%	

*Negative results in the simulation output SUM column indicate a negative effect of the parameter and vice versa. The baseline scenario covers all patients for the entire duration of the disease. The last 5 years scenario focuses on the last 5 years; the number of patients in the cohort is reduced by one because the disease, in that case, lasted only 1 year. The medicated only scenario describes the set of medicated patients in the last 5 years of disease duration, regardless of the length of medication and clinical outcomes*.

The table of simulation results shows that the parameters that influence the patient's experience of the disease most include sleep drunkenness, the refreshing potential of naps, the quality of nocturnal sleep, and the social aspects of the patient's life. Psychiatric conditions influence the overall pattern at a low level. Medication had a similarly low contribution to the overall dynamics.

**Figure 2:**
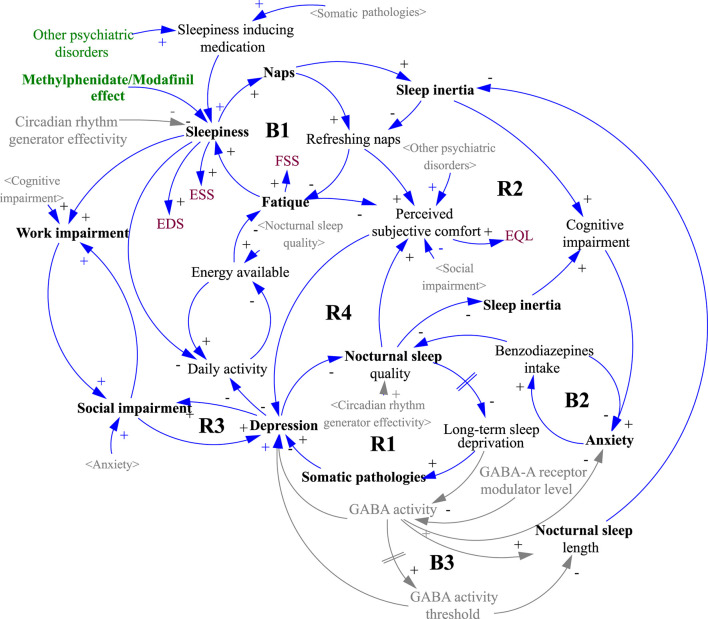
Causal loop diagram of the simulation model structure. The gray variables in sharp brackets < > are copies of the black originals and are used to increase the clarity of the diagram. The gray variables without brackets and the gray causal links were not included in the simulation, indicating a possible direction for further model development. A detailed description of the diagram logic is given in the Results section.

The underlying structure (causal loop diagram) of the model and the starting point for the simulations is depicted in Figure 2. The mathematical signs for causal links in the form of arrows express the tendency of the causal coupling of variables, where the origin of the arrow comes from the influencing variable and the arrowhead points toward the influenced variable. A positive sign indicates a coincident tendency, where an increase in the influencing variable leads to an increase in the controlled variable. A negative sign indicates the opposite binding tendency, where an increase in the value of the influencing variable causes a decrease in the value of the influenced. It should be stressed that the relationships are nonlinear, and so the magnitude of change cannot be inferred from the number of input links. For example, a number of linkages entering into the variable 'depression' do not necessarily imply the highest rate of change or the highest contribution to the overall dynamics. The role of the system diagram and model is to describe all known linkages, even though some or all may have relatively marginal effects. Another characteristic of a system diagram is the ability to express feedback loops, which are of two kinds. Positive feedback loops are denoted by R (reinforcing) and negative feedback loops by B (balancing). Positive feedback loops tend to induce a rapid change of state, often of the nature of an exponential increase or decrease, while negative loops lead the system to a steady-state but also inhibit possible evolution.

In terms of the overall dynamics, which of the feedback loops is dominant in the system is decisive. However, loop dominance may not be a constant in natural systems. Often a shift of dominance occurs when the initially dominant loop loses its power to another. In Figure 1, this phenomenon is particularly evident at the point marked (b), where the original growth activity is halted by the action of the newly dominant negative feedback loop.

## Discussion

A number of papers on IH include sets of symptoms and possible causes and analyses of the impact of the disease on the patient's life. In most cases, these are presented as bulleted lists or tables or continuous text listing factors (15, 25, 26). Papers have also been published in which putative relationships have been shown in the form of various diagrams, and one cited even suggests the feedback nature of fatigue and sleep (11, 20). This work is based on a systems view of the problem and works with a dynamic model consisting of a system of differential equations (27, 28). The role of the model is to capture the dynamics of the disease from the patient's perspective and compare the patient's experience of the disease course with objective data obtained during a comprehensive, detailed interview. We assume that if the fit between the patient's view of the course of the disease and the simulated influence of the objective parameters determining the patient's experience is high, the model can be considered acceptable, and the contribution of the individual influences to the overall experience can be inferred from the simulation results. The model simulates all patients individually and yet simultaneously because its structure uses a matrix. By simulating the individual runs in the manner described in the results section, a fit between patient testimony and simulated objective parameters was established. Subsequently, the results of all patients were summarized, and an overall fit between the reported and simulated waveforms of 76.1% was found. As the value obtained is in the highest quartile, we consider it acceptable for drawing further conclusions (29).

From the results, it may seem that the role of medication is quite marginal, but it is important to note that results cover the entire duration of the disease for all patients, thus reaching times when medication was not even available for all subjects due to formal reasons (30). In a paper published last year by Maski et al. (31), the authors report that clarithromycin has only a partial effect on the treatment of IH, flumazenil has virtually no effect (those drugs were not used), as well as methylphenidate (which our patients took), modafinil has a slightly better effect, and pitolisant and sodium oxybate have a partial effect (the last 2 drugs were again not used in this study). Overall, the authors conclude that the effect of current medical therapy on IH is insufficient. Although the values for the proportion of medication are also low in the last two scenarios, at first sight, its role doubles for modafinil in the medicated group compared to the whole group. However, the direct contribution to the overall dynamics cannot be read in the same way as the conventional statistics (32).

Dynamic simulation introduces a time dimension compared to a more or less sophisticated statistical analysis based on one static “snapshot.” For example, a static view of the effect of modafinil may answer the question of how modafinil over time affects the overall nonlinear dynamic interplay of all factors that lead to patients' perceptions of the impact of IH, but it does not answer the question of the relative contribution of modafinil to patients' perceptions at a particular point in time. Interestingly, a comparison of the “last 5 years” and “last 5 years medicated only” scenarios shows relatively little difference in the impact of medication but a significant difference in the overall profile, for example, in the effect of work and social disability (15.2 vs. 8.5%). The change in the overall profile may provide valuable clues for formulating a rational treatment and prophylactic strategy.

It is important to note that the contribution of each parameter to the overall patient experience of the disease is not linear and follows a structure, as shown in Figure 2. That structure is depicted as a systemic causal loop diagram but cannot be considered complete since it only includes the variables shown in black font. The gray shading is included to illustrate the overall picture and is based on review articles and works cited, in particular the potential role of circadian rhythm generator efficiency in the review paper by Billiard and Sonka (5) and the role of GABA in articles by Dauvilliers et al. (33), Trotti (34) and Trotti et al. (35).

Results indicate that the contribution of each parameter varies between scenarios. The parameters that most significantly affect the dynamics are those belonging to the basic disease picture, among which sleep inertia, naps, and quality of night sleep stand out (5, 34, 36). At first glance, it may seem that the difference between the whole group and the medicated patients is not noticeable in some parameters. However, a comparison of the simulated data demonstrates in the last column (*p*) that the influence of social and work impairment, sleepiness, naps, nocturnal sleep, and somatic pathologies differ significantly. The model simulation results confirm the conclusions of previous work. Thus, it can be speculated that the present dynamic structure at least partially reflects the conditions in the system that manifests as IH (12, 15, 26). Surprising was the low contribution rate for psychiatric disorders, which are outweighed by the resulting work and social influences (1, 37, 38). Twelve patients (27%) were diagnosed with depression in the whole cohort. Anxiety was diagnosed in six, but only one did not have comorbid depression. However, the BDI values at the time of the interview in these patients (although this is a self-report screening, it is widely used in clinical context because of a high internal consistency (Cronbach's alpha = 0.91) performed by Beck et al. (39) and a high correlation with objective tools, as e.g., Hamilton Depression Scale, r=0.71 by Steer et al. (40) suggest that in all cases, this was a therapeutically well-managed affective disorder. The end-point score was 7 in the whole cohort and 13.3 in those diagnosed with depression, which is below half the cut-off score for severe depression (0–13 minimal depression, 14–19 mild depression, 20–28 moderate depression, and 29–63 severe depression). Despite the high prevalence of comorbid depression, this condition seems to be well-managed in most patients. Its overall impact in the model is attenuated by other non-psychiatric factors, with none of few treatment options.

It is necessary to state that this study has significant limitations. It is based on a model, and any model is, by definition, an inaccurate approximation of the reality represented. While the selection of inputs is an intersection of those repeatedly found in works on IH, some, given the general level of knowledge, might be missing. If there are advances in understanding the role of parameters not yet included, the model can be, for example, extended to incorporate a circadian activity generator or GABA. The correctness of choice and the setting of the model equations were verified by comparing the patient's testimony with the simulation output, and the result obtained is not 100%. Although the results show which parameters play the most prominent role in the overall patient experience of the disease and the set corresponds to the clinical experience in previously published articles, their exact, realistic contribution cannot be verified at the present time. Despite the considerable size of the input data, there are imperfections in the dynamic description of the parameters. Thus, the input data represent a compromise between methodological requirements and a tolerable patient interview burden. For some input parameters, it may be possible to obtain more reliable, objectively measured data using advanced diagnostic methods (41). Similarly, the role of influences not captured by the model remains at the hypothesis level. Further research will be needed to develop a more refined model with a broader input database, providing deeper insight into IH dynamics.

## Conclusion

From a clinical perspective, we consider the finding of a relatively small proportion of psychiatric comorbidities and the effect of medication on the overall experience of illness to be significant. It should be emphasized that some influence on the overall dynamics may also be due to the state prescription policy, which in our country allows off-label administration of stimulants for IH patients, and after approval by the reviewing physician of the respective health insurance company. Nevertheless, the dominant parameters influencing patient experience identified by the model suggest in which direction therapeutic efforts should be directed. Difficult-to-control factors such as sleep inertia are the most prominent, but thought can be given to improving the availability of stimulants and psychotherapeutic interventions targeting patients' work and social impairment. Although each model must be considered a partial and imperfect representation of reality, even the simplified dynamic structure designed by the research team reflects the real-life events in patients with IH at the acceptable level of 76.1% and suggests that a similar structure plays an essential role in the course of the disease. Therapeutic focus on the parameters identified by the model should enhance the patients' subjective experience throughout illness duration and might even turn the progress from negative into positive. It is hoped that future work will answer both the question of disease etiopathogenesis and the optimal therapeutic approach, and this simulation model will help reach these goals.

## Data Availability Statement

The raw data supporting the conclusions of this article will be made available by the authors, without undue reservation.

## Ethics Statement

The studies involving human participants were reviewed and approved by Ethics Committee of the First Faculty of Medicine, Charles University, Prague, CZ. The patients/participants provided their written informed consent to participate in this study.

## Author Contributions

MS: questionnaire development, model development, statistical processing of results, and manuscript. SN and KŠ: questionnaire development, data collection, model development, statistical evaluation of results, and manuscript. SP and GB: model building, statistical evaluation of results, and manuscript. All authors contributed to the article and approved the submitted version.

## Funding

This research was supported by the Czech Ministry of Health, Health Research Agency Grant Number NU20-04-00088, the Charles University Cooperatio MED/Neuroscience program, and Proverbs grant NEURO2021-2.

## Conflict of Interest

The authors declare that the research was conducted in the absence of any commercial or financial relationships that could be construed as a potential conflict of interest.

## Publisher's Note

All claims expressed in this article are solely those of the authors and do not necessarily represent those of their affiliated organizations, or those of the publisher, the editors and the reviewers. Any product that may be evaluated in this article, or claim that may be made by its manufacturer, is not guaranteed or endorsed by the publisher.
